# Fatal myocardial infarction investigated using contrast‐enhanced postmortem computed tomography: A case report

**DOI:** 10.1002/ccr3.8340

**Published:** 2023-12-18

**Authors:** Kohei Aoe, Yuichi Orita, Chikage Oshita, Shuji Date, Hiroki Teragawa

**Affiliations:** ^1^ Department of Clinical Education JR Hiroshima Hospital Hiroshima Japan; ^2^ Department of Cardiovascular Medicine JR Hiroshima Hospital Hiroshima Japan; ^3^ Department of Radiology JR Hiroshima Hospital Hiroshima Japan

**Keywords:** acute ST‐elevation myocardial infarction, autopsy imaging, cardiac tamponade, contrast enhanced postmortem computed tomography

## Abstract

Conventional autopsies are considered standard methods for clarifying cause of death. However, because of the increasing use of computed tomography, magnetic resonance imaging, and other diagnostic imaging techniques, autopsy imaging is now more frequently adopted to identify diseases with unknown causes and sudden deaths. A 84‐year‐old man was diagnosed with acute myocardial infarction using coronary angiography. After taking oral antiplatelet medication in the catheterization laboratory, the patient suddenly coughed violently, lost consciousness, and was diagnosed with cardiac arrest. Spontaneous circulation did not return after 50 min of cardiopulmonary resuscitation. To elucidate the cause of the cardiac arrest, we performed contrast‐enhanced postmortem computed tomography (PMCT), which revealed cardiac tamponade due to cardiac rupture of the inferior myocardium. Our findings reaffirm the effectiveness of contrast‐enhanced PMCT in the diagnosis of sudden death in the clinical setting.

## Graphical abstract

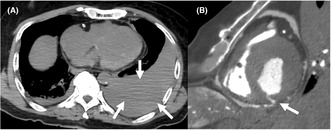

## 1 INTRODUCTION

Autopsies are performed to confirm clinical findings, provide more complete information regarding the cause of fatality, or uncover conditions that were not recognized before death.^1^ Autopsy rates are declining worldwide and there are two main reasons for this: first, the autopsy rate is high for deaths due to external reasons such as trauma and fewer people die of external causes in today's graying society.^2^ Second, the advent of modern imaging techniques, such as computed tomography (CT) or magnetic resonance imaging, may have contributed to the lower rate of autopsies performed.^3^ Autopsy imaging (AI) can be used as an alternative to traditional autopsies.

Herein, we report a case in which cardiac arrest occurred immediately after coronary angiography in the evaluation of acute myocardial infarction (AMI) and discuss the process of elucidating the cause of death through contrast‐enhance postmortem CT (PMCT), which is a type of AI.

## 2 CASE PRESENTATION

**FIGURE 1:**
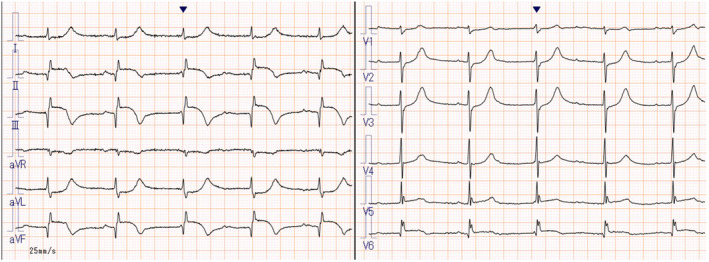
Electrocardiogram (ECG) at admission. ECG at admission showing a complete A‐V block with abnormal Q waves and ST elevations in the II, III, and aVF leads.

A 84‐year‐old man presented to our hospital complaining of left flank pain, vomiting, and anorexia that persisted for 24 h. The patient was 161 cm tall, weighed 63 kg, and had a body mass index (BMI) of 24.3 kg/m^2^. His consciousness was not impaired, and his blood pressure was 123/84 mmHg with a pulse of 61 beats/min. The oxygen saturation on room air was 94%, and his body temperature was 37.2°C. On auscultation, a heart sound was irregular with variable intensity; however, no murmurs were heard. The lung fields were clear, and no rales were heard. Computed tomography (CT) showed cholecystitis but no pleural effusion. However, blood tests showed elevations of creatine kinase (CK) at 4049 U/L, CK‐MB at 336 U/L, and troponin T at 14.621 ng/mL. An electrocardiogram (ECG) showed a complete A‐V block with abnormal Q waves and ST elevations in leads II, III, and aVF (Figure 1). We examined his heart by transthoracic echocardiography (TTE) and found dyskinesis involving the inferior myocardium. An eyeball assessment of the left ventricular ejection fraction (LVEF) was approximately 40%. We suspected AMI and performed emergency coronary angiography (CAG) for treatment of the occluded coronary artery.

**FIGURE 2:**
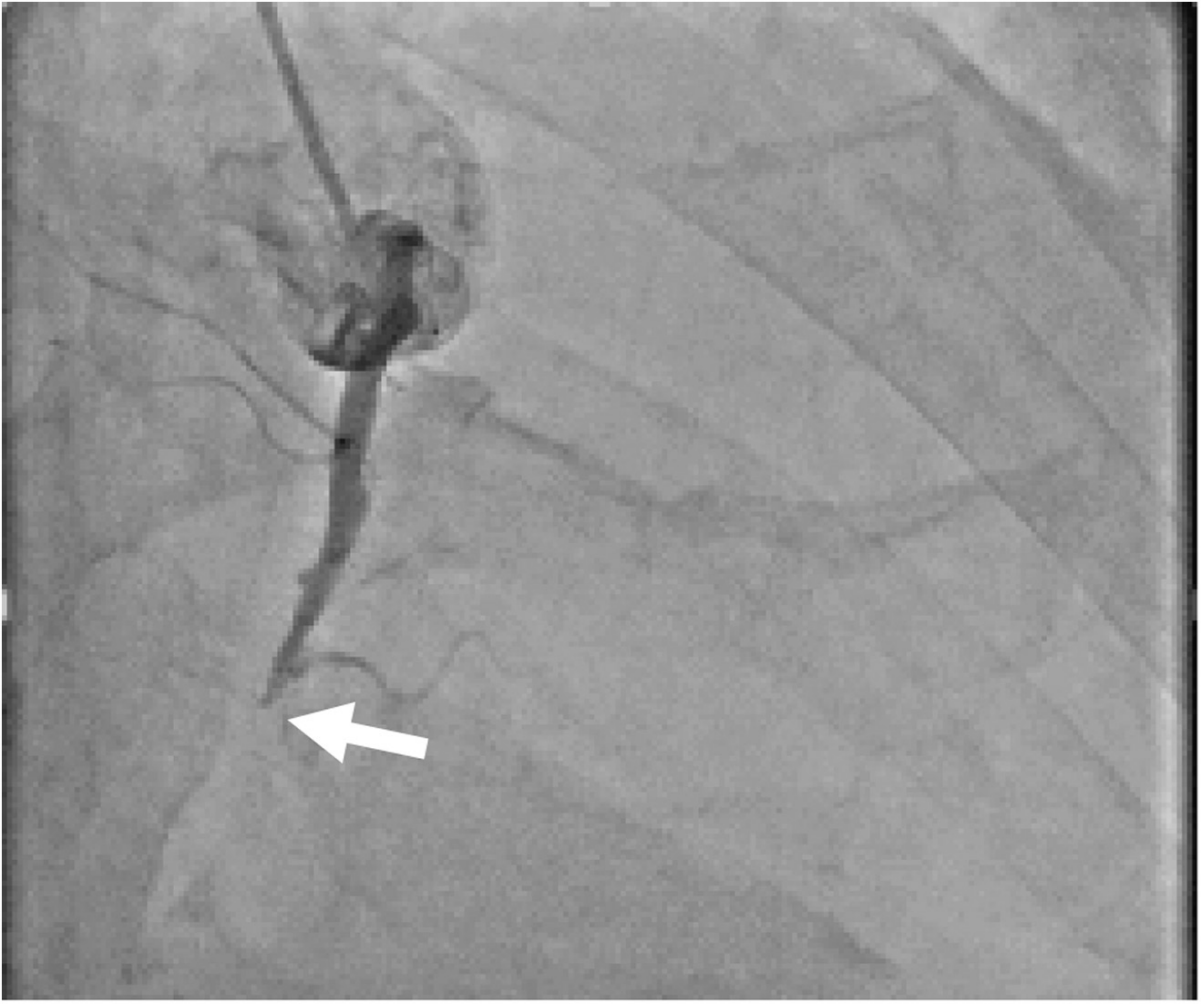
Coronary angiography (CAG). CAG shows the occlusion (arrow) at the mid‐portion segment of the RCA. CAG, coronary angiography; RCA, right coronary artery.

CAG revealed occlusion at the midportion of the right coronary artery (RCA) (Figure 2), and the preparations for percutaneous coronary intervention (PCI) were started. The patient suddenly coughed violently after taking prasugrel hydrochloride tablets. We suspected aspiration of the drug or anaphylaxis of the contrast agent and injected chlorpheniramine maleate and prednisolone. Immediately thereafter, he lost consciousness. The diagnosis of pulseless electrical activity was based on electrical activity on monitor with no pulsation. Cardiopulmonary resuscitation (CPR) was initiated. During CPR, TTE showed pericardial and pleural effusion. The amount of pericardial effusion was small, and cardiac arrest was not recognized as cardiac tamponade at that time, and pericardiocentesis was not performed. We also attempted to insert an intra‐aortic balloon pump and extracorporeal membrane oxygenation through the femoral vessels. However, the arterial cannulation failed due to the significant peripheral vascular disease on either side. The arteries of the bilateral lower extremities showed stenotic lesions with severe calcification on echo, which prevented insertion of the guidewire. After 50 min of CPR, his spontaneous circulation never returned, and CPR was terminated. To elucidate the cause of the cardiac arrest, we obtained permission to perform contrast‐enhanced PMCT from his family.

**FIGURE 3:**
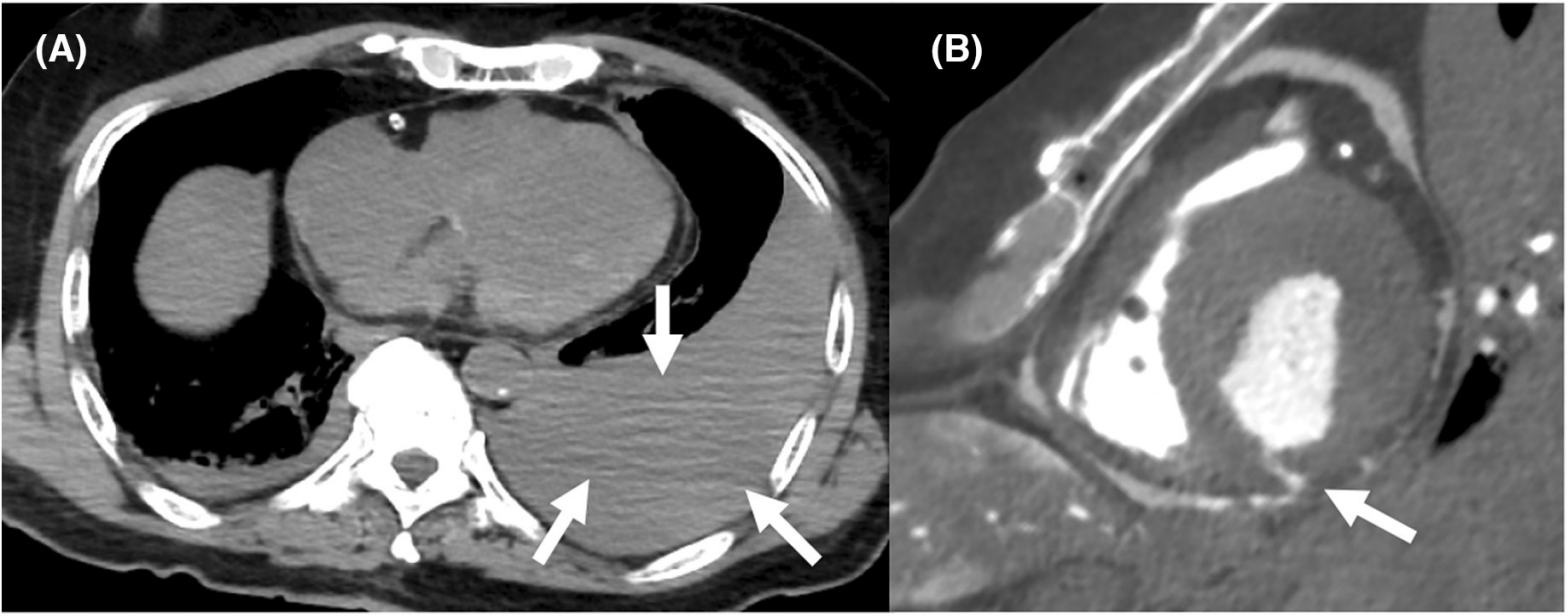
Autopsy imaging (AI). (A) Plain CT shows massive pleural effusion (arrows) of 50 HU on AI, which implied that the fluid was blood. (B) After a few sets of chest compression, CT showed the contrast agent flow (arrow) from the cardiac cavity into the pericardium. AI, autopsy imaging; CT, computed tomography; HU, Hounsfield unit.

**FIGURE 4:**
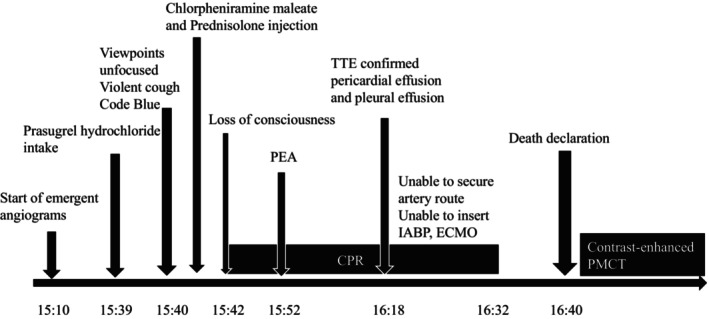
Time course of the present case. CPR, cardiopulmonary resuscitation; ECMO, extracorporeal membrane oxygenator; IABP, intra‐aortic balloon pumping; PEA, pulseless electrical activity; PMCT, postmortem computed tomography; TTE, transthoracic echocardiography.

Plain CT showed massive pleural effusion of 50 Hounsfield units (HU), implying that the fluid was blood (Figure 3A). A contrast agent was then injected into the body through the right internal jugular vein at a volume of 120 mL, followed by 30 chest compressions. After a few sets of compressions, CT showed contrast agent flow from the cardiac cavity into the pericardium (Figure 3B), indicating a strong possibility of cardiac tamponade. We further compressed the chest; however, we could not observe contrast agent flow from the pericardium into the pleural cavity. Although there was no 100% certainty, we determined that the immediate cause of death was cardiac tamponade caused by perforation of the inferior wall of the left ventricle, likely due to a violent cough at the necrotic myocardium caused by acute inferior infarction. The time course of the case is shown in Figure 4.

## 3 DISCUSSION AND CONCLUSION

We encountered a case of a patient with AMI of the inferior wall who went into cardiac arrest after taking prasugrel hydrochloride in preparation for PCI; PMCT demonstrated cardiac tamponade following cardiac rupture. Cardiac tamponade following cardiac rupture was thought to be the immediate cause of death, considering the clinical fact that the patient coughed violently after taking medication.

According to a previous study,^4^ early rupture (within 72 h) of the left ventricle is more likely to occur at anterior infarction sites. Another study^5^ found that myocardial rupture was 9.2 times more likely to occur in patients with all of the following characteristics: no history of previous angina or myocardial infarction, ST‐segment elevation or Q wave development on the initial ECG, peak CK‐MB above 150 U/L. The ruptured part was at the inferior infarction site in the present case, and this case met the aforementioned risk factors. Furthermore, in another epidemiological study,^6^ predictors of cardiac rupture after myocardial infarction included female sex, low LVEF (<40%), high heart rate (≥94/min), low BMI (<25 kg/m^2^), and older age (age≧68 years). Although this patient was male and his heart rate was low, which was complicated by a complete A‐V block, he met the other three criteria. In conjunction with the aforementioned risk factors, it is necessary to be aware that this patient was at a high risk of cardiac rupture. Generally speaking, the prevention of an increase in abdominal pressure, such as defecation control, is recommended in the acute care of AMI,^7^ because it is possible that such pressure can cause cardiac rupture. In addition, it has been reported that the intrathoracic pressure transiently increases to 100–250 mmHg due to violent cough.^8^ However, in this case, the association between medication intake and violent coughing with cardiac rupture was not initially recognized highlighting the importance of preventing increased intrathoracic or abdominal pressure to reduce the risk of myocardial rupture.^7^, ^8^

The usefulness of PMCT in clarifying the cause of death has previously been reported.^9^, ^10^, ^11^ One of these reports prospectively compared the AI with clinical diagnoses.^10^ The report showed a discrepancy between AI and clinical determination of the immediate cause of death; accuracy rates were 46% for clinical diagnosis and 74% for AI. In another prospective study, a major discrepancy existed between autopsy and imaging causes of death in 30% of the cases.^12^ Contrast agents were not used in these studies. Therefore, there are no data on the differences in the accuracy of PMCT between contrast‐enhanced CT and plain CT. We were able to confirm rupture of the infarcted cardiac muscle of the inferior wall and contrast agent flow from the left ventricle into the cardiac cavity, which strongly supports the idea that cardiac tamponade following cardiac rupture was the immediate cause of death, although there is no 100% certainty. As shown in the present case, PMCT using contrast agents may be more useful in the cause of death in the cardiovascular field. Furthermore, severe hemothorax on the left side without any mediastinal shift was considered to be the results of damage to the intercostal arteries or an internal mammary artery caused by chest compression, and/or of blood leaking from the pericardial sac into the thoracic cavity although contrast‐enhanced PMCT did not demonstrate a clear flow of blood from the pericardial effusion into the thoracic cavity. We do not believe that pleural effusion is a direct cause of death; however, the large volume may have had a negative effect on the course in this case. The pros and cons of performing contrast‐enhanced PMCT in this case are summarized again. The cons are the time and medical cost issues. Since this was a highly disabling inferior wall infarction in terms of myocardial deviation enzymes, complications including cardiac rupture were a possibility. The advantage of this modality, however, was that it was able to clarify the cause of the cardiac arrest. It is important to be able to explain the cause of sudden death to the patient's family. For medical staffs, the advantages outweighed the disadvantages: we were able to reaffirm how to deal with cardiac rupture even in the case of inferior wall infarction, and we were able to redefine hospital rules such as the timing and safe administration of antiplatelet agents.

Despite the usefulness of PMCT with contrast agents, there is no standardized protocol; the protocol used depends on the examiner. Therefore, we injected 120 mL of contrast agent, which was the empirically determined dosage at our hospital, through the right internal jugular vein. We performed three sets of chest compressions 30 times to ensure that the pulmonary arteries, heart, coronary arteries, and blood vessels throughout the body, including the brain, were contrasted at each time phase. The amount and site of contrast agent administration, the number of chest compressions, and the number of imaging sessions were dependent on the facility. Thus, it is necessary to consider more cases of PMCT using contrast agents to develop better protocols.

In conclusion, we encountered a case of AMI of the inferior wall that developed into cardiac tamponade following cardiac rupture while preparing for PCI. We identified the cause of the patient's death using contrast‐enhanced PMCT. Based on this case, there are several aspects to reflect on, and it is important to identify cases with a strong possibility of cardiac rupture for appropriate treatment. Contrast‐enhanced PMCT is an important tool for identifying the cause of cardiac arrest in the cardiovascular field, and it is important to amass cases to standardize contrast‐enhanced PMCT protocols.

## AUTHOR CONTRIBUTIONS

**Kohei Aoe:** Data curation; writing – original draft. **Yuichi Orita:** Data curation; investigation; writing – original draft; writing – review and editing. **Chikage Oshita:** Data curation; writing – review and editing. **Shuji Date:** Data curation; methodology; writing – review and editing. **Hiroki Teragawa:** Data curation; investigation; writing – review and editing.

## FUNDING INFORMATION

The authors received no financial support for the research, authorship, or publication of this manuscript.

## CONFLICT OF INTEREST STATEMENT

The authors declare no potential conflicts of interest with respect to the research, authorship, and/or publication of this article.

## ETHICS STATEMENT

This study was conducted in accordance with the 1964 Declaration of Helsinki and its amendments.

## CONSENT

Written informed consent was obtained from the patient's family for the publication of this case report and accompanying images.

## Data Availability

Research data are not shared.
